# Supplementary material for: Parkin-dependent mitophagy occurs via proteasome-dependent steps sequentially targeting separate mitochondrial sub-compartments for autophagy
Source: Autophagy Rep. 2022 Dec 19;1(1):576–602. doi: 10.1080/27694127.2022.2143214 (PMC11864704; doi:10.1080/27694127.2022.2143214)
Supplement: Supplemental Material [file KAUO_A_2143214_SM2097.zip › StepwiseMitoAutophagyReportRefinal_rev13102022.docx]

**Parkin-dependent mitophagy occurs via proteasome-dependent steps sequentially targeting separate mitochondrial sub-compartments for autophagy**

Anna Lechado-Terradas^1,2^, Sandra Schepers^1^, Katharina I. Zittlau^3^, Karan Sharma^2,4^, Orkun Ok^1,2^, Julia C. Fitzgerald^4^, Stefan Geimer^5^, Benedikt Westermann^5^, Boris Macek^3^ and Philipp J. Kahle^1,2,6^

^1^Laboratory of Functional Neurogenetics, Department of Neurodegeneration, Hertie Institute for Clinical Brain Research, Faculty of Medicine, University of Tübingen, Tübingen, Germany; ^2^Department of Biochemistry, Faculty of Science, University of Tübingen, Tübingen, Germany; ^3^Quantitative Proteomics Group, Department of Biology, Interfaculty Institute of Cell Biology, University of Tübingen, Tübingen, Germany; ^4^Mitochondrial Biology of Parkinson’s Disease, Department of Neurodegeneration, Hertie Institute for Clinical Brain Research, Faculty of Medicine, University of Tübingen, Tübingen, Germany; ^5^Laboratory of Cell Biology and Electron Microscopy, University of Bayreuth, Bayreuth, Germany, ^6^German Center for Neurodegenerative Diseases, Tübingen, Germany

**CONTACT**

[boris.macek@uni-tuebingen.de](mailto:boris.macek@uni-tuebingen.de); philipp.kahle@uni-tuebingen.de

**KEYWORDS**

mitochondrial outer membrane; mitophagy; parkin; proteasome; ubiquitin

**ABSTRACT**

PINK1/parkin-dependent mitophagy initially involves (phospho)ubiquitin-directed proteasome-dependent degradation of certain outer mitochondrial membrane (OMM) proteins (e.g. mitofusins) and the recruitment of autophagy effectors to a group of ubiquitinated OMM proteins, eventually leading to autophagic removal of damaged mitochondria in stressed cells. Here we provide evidence that mitochondrial degradation occurs via stepwise delivery of separate mitochondrial sub-compartments for autophagic degradation. OMM and inner mitochondrial material appears to become separately isolated for autophagolysosomal degradation, not only in parkin-overexpressing HeLa cells but also in cells that express endogenous parkin (human embryonic kidney cells and neural progenitor cells) with slower mitophagy kinetics. The remaining inner mitochondrial material becomes degraded only after much prolonged membrane depolarization, potentially involving another proteasome-sensitive step. The present combined microscopy and proteomics analyses support the idea that cell stress-induced parkin-dependent mitophagy is a complex multi-step process with distinct mitochondrial sub-compartments being separately targeted for autophagic degradation.

**Abbreviations:** BafA, Bafilomycin A; CCCP, carbonyl cyanide 3-chlorophenylhydrazone; COX IV, cytochrome c oxidase subunit IV; CS, citrate synthase; DMEM, Dulbecco’s modified Eagle’s medium; EGFP, enhanced green fluorescent protein; FBS, fetal bovine serum; IF, immunofluorescence; IMM, inner mitochondrial membrane; KO, knock-out; LC3, microtubule-associated protein 1 light chain 3; MDVs, mitochondria-derived vesicles; MFN, mitofusin; NPCs, neural progenitor cells; OMM, outer mitochondrial membrane; p62/SQSTM, 62kDa protein sequestosome-1; PBS, phosphate-buffered saline; PINK1, phosphatase and tensin homolog (PTEN)-induced putative kinase protein 1; RT, room temperature; SSBP1, single-stranded DNA binding protein 1; TAX1BP1, Tax1-binding protein 1; TEM, transmission electron microscopy, TOM20, translocase of outer mitochondrial membrane 20kDa subunit; TOM70, translocase of outer mitochondrial membrane 70kDa subunit; Ub, ubiquitin; UPS, ubiquitin proteasome system; VDAC, voltage-dependent anion-selective channel protein; WB, Western blot; WT, wild-type

**Introduction**

Acute mitochondrial stress conditions trigger the specific autophagic elimination of mitochondria in a process termed mitophagy. One of the most studied forms of mitophagy is the one orchestrated by the phosphatase and tensin homolog (PTEN)-induced putative kinase protein 1 (PINK1) and the E3 ubiquitin-protein ligase parkin [1]. Mutations in both proteins have been linked to the most common recessive hereditary forms of Parkinson’s disease and are known to compromise successful mitophagy [2–5].

There are several ways to experimentally induce PINK1/parkin mitophagy [6]. One of the most widely used is treatment with the uncoupler carbonyl cyanide 3-chlorophenylhydrazone (CCCP), which promotes proton permeability of the inner mitochondrial membrane (IMM), harshly inducing mitochondrial depolarization [7]. When modeling mitophagy in neuron-like cell models that strongly rely on mitochondrial respiration, dissipation of the mitochondrial membrane potential might not be enough to promote mitophagy [6]. In neural cells mitophagy can be induced by respiratory complex III inhibition with Antimycin A [8,9].

Under depolarizing conditions, the mitochondrial protein import machinery cannot translocate PINK1 across the membranes and thus PINK1 accumulates at the outer mitochondrial membrane (OMM) [10]. There, PINK1 phosphorylates cytosolic ubiquitin (Ub) and the parkin Ub-like domain, both at Ser65 [11,12]. Such parkin phosphorylation stimulates its ubiquitin ligase activity and translocation to the OMM, where parkin strongly binds to phosphorylated ubiquitin (pS65-Ub) at its active center [13]. At this point, parkin assembles pS65-Ub chains on OMM substrates, which either trigger rapid degradation by the ubiquitin-proteasome system or promote the binding of autophagy adaptors [14–16].

Parkin-dependent mitochondria elimination involves the binding of ubiquitinated OMM proteins by autophagy adaptors, which specifically recognize ubiquitinated cargo through their ubiquitin binding domain and link ubiquitinated mitochondria to the autophagic membrane through their microtubule-associated protein 1 light chain 3 (LC3)-interacting region. Recognition of ubiquitinated mitochondria by autophagy adaptors promotes the engulfment of mitochondria in autophagosomes that eventually fuse with lysosomes for wholesale degradation [17,18]. However, there is recent evidence for selective sub-mitochondrial degradation pathways in yeast stationary-phase mitophagy [19].

Our most recent study on PINK1/parkin-dependent temporal ubiquitination and phosphorylation events indicated a stepwise mitochondrial degradation in wild-type parkin (WT-parkin) expressing HeLa cells [20]. Here, we examined the stepwise mitochondrial degradation mechanisms in greater detail and discuss the specific involvement of proteasome and autophagy mechanisms that influence PINK1/parkin mitophagy at early and late stages of the pathway. We provide evidence that OMM and IMM material is separately delivered to the lysosomal machinery, suggesting distinct degradation pathways for individual mitochondrial sub-compartments. This was not only observed in parkin-overexpressing HeLa cells, but also in cells with endogenous parkin expression levels (human embryonic kidney HEK293 cells and neural progenitor cells (NPCs)) that showed slower mitophagy kinetics. In addition to the OMM proteasome dependence for mitophagy initiation, we provide evidence for a potential second proteasome-sensitive IMM regulatory step for mitophagy elimination of matrix proteins. The present integrative microscopy and proteomics analyses indicate that PINK1/parkin-dependent mitophagy is a multi-step process modulated at several levels by the proteasome, where distinct mitochondrial sub-compartments are separately targeted to autophagic degradation with distinct kinetics.

**Results**

***Distinct subsets of OMM proteins are subject to immediate-early proteasomal and delayed autophagic degradation***

When WT-parkin HeLa cells are exposed to the mitochondrial membrane uncoupler CCCP, mitochondria are subjected to PINK1/parkin-dependent degradation. Interestingly, the degradation of mitochondrial proteins does not take place at once, but rather follows a stepwise mitochondrial sub-compartment degradation dynamics [20]. OMM proteins are the first ones to be degraded, followed by IMM and matrix proteins. For example, mitofusin-1 (MFN)-1 was extremely rapidly degraded with a half-life time of <2 h (Figure 1A). The rapid removal of parkin-ubiquitinated MFN proteins occurs via direct proteasomal targeting [21]. Indeed, pretreatment with the proteasome inhibitor MG132 caused an accumulation of ubiquitinated MFN1 after 4 h CCCP treatment (Figure S1A).

Other OMM proteins such as voltage-dependent anion-selective channel (VDAC), translocase of outer mitochondrial membrane 70 kDa subunit (TOM70) and 20 kDa subunit (TOM20) had more delayed degradation rates with half-life times of 2-4 h (Figure 1A). At 4 h CCCP treatment, TOM20-immunopositive structures became devoid of the early proteasome targets MFN1 and TOM70 (Figure S1C and S1D), indicating engagement of delayed OMM substrates in mitophagy after the earlier proteasome substrates were removed. The IMM protein cytochrome c oxidase subunit IV (COX IV) had a slightly slower degradation rate, showing a half-life time of ≈4 h. However, the degradation of matrix proteins citrate synthase (CS) and single stranded DNA binding protein 1 (SSBP1) was hardly detectable at 4 h CCCP treatment, their half-life times being >8 h (Figure 1A). Indeed, when most of the OMM proteins were removed after 14 h (Figure 1A), remaining inner mitochondrial markers (SSBP1, COX IV) persisted while at this time point the TOM20 remnants were reduced to puncta (Figure 1B).

Pretreatment with proteasome inhibitors stabilized MFN1 and TOM70 also as higher molecular mass species (Figure S1A and S1B), indicating direct proteasome-targeting ubiquitinations for these rapidly degraded OMM proteins. In contrast, although the slower degradation of TOM20 and VDAC was likewise prevented by proteasome inhibitor pretreatment, this was not accompanied by an accumulation of ubiquitinated species (Figure S1A and S1B). The slower removal of such OMM proteins may therefore not occur through direct ubiquitin-dependent proteasome degradation, but is mediated after an initial proteasome-dependent step (see below). Degradation of inner mitochondrial proteins (COX IV, CS) was also suppressed after pretreatment with proteasome inhibitors (Figure S1A and S1B). Thus, pretreatment with proteasome inhibitors blocks the removal of direct substrate proteins on the OMM and prevents an important early proteasome-dependent step for the initiation of mitophagy in CCCP-treated parkin-HeLa cells. Putative not proteasome-targeting ubiquitinations of VDAC and TOM20 that can be seen upon long exposure are in fact reduced after pretreatment with MG132 (Figure S1A). Such ubiquitinations can be visualized on TOM20-positive and SSBP1-positive structures in the course of mitophagy (Figure 1C), evidently recruiting ubiquitin-binding autophagy receptors such as the 62 kDa protein sequestosome-1 (p62/SQSTM) (Figure 1D) and Tax1-binding protein 1 (TAX1BP1) (Figure 1E).

The recruitment of ubiquitin-binding autophagy receptors should connect to the autophagy machinery via ATG8 proteins. Immunostaining of endogenous LC3B confirmed an association of TOM20 puncta after 8 h of mitochondrial depolarization (Figure S1E). Most TOM20 puncta (dissociated from SSBP1-positive mitochondrial structures) were in close apposition with LC3B puncta. At this time point, endogenous LC3B puncta also came in apposition to COX IV positive structures, while SSBP1-positive aggregates did not (yet) co-localize with endogenous LC3B (Figure S1E). Endogenous LC3B-positive autophagic puncta were dispersed throughout the cells with little enrichment on mitophagy cargo, perhaps indicating some limitation of the anti-LC3B immunostaining. Thus, transfection experiments with EGFP-LC3B were performed, which showed somewhat more prominent translocation to mitophagy cargo (Figure 1F). Consistent with the immunostaining results for endogenous LC3B, EGFP-LC3B was associated with most TOM20-only puncta (Figure 1F), either showing close apposition (arrows) or complete overlap (closed arrowheads). Rare EGFP-LC3B-negative TOM20-only puncta were observed (open arrowheads). Such features might represent mitochondria-derived vesicles (MDVs), which were described to be delivered to lysosomes independently from LC3 [22]. Alternatively, EGFP-LC3B-negative puncta could simply reflect TOM20 cargo that has not yet engaged LC3B phagophores at this time point. As for endogenous LC3B, the EGFP-tagged LC3B showed very little overlap with SSBP-1 positive aggregates devoid of OMM markers at this time point (Figure 1F, ROI 1), but some SSBP1-LC3B positive puncta started to become detectable (Figure 1F, ROI 2, arrows).

Autophagy completion depends on the formation of autophagosomes that are eventually fused with lysosomes, inside of which the targeted cargo is degraded by acidic hydrolases [17,23]. Indeed, as mitophagy progressed, the TOM20 puncta came to increasingly close apposition with the lysosomal-associated membrane protein 1 (LAMP1) (Figure S1F), suggesting cargo delivery to lysosomes [24]. The active engagement in autophagolysosomes was confirmed with LysoTracker RED (Figure 1G), a fluorescent marker that can be used for monitoring lysosome acidification [24,25]. When lysosomal acidification as well as autophagosome-lysosome fusion was inhibited by pretreatment with Bafilomycin A (BafA), the number of TOM20-positive as well as SSBP1-positive puncta significantly increased after 8 h of CCCP treatment (Figure 1H and I), indicating that the mitochondrial material engaged in (autophagic) puncta was eventually delivered to lysosomes.

Additionally, in order to confirm that the degradation of mitochondrial sub-compartment proteins did rely on autophagy machinery targeting, HeLa cells lacking all autophagy adaptors (penta knock-out cell line, referred as pentaKO) [16] were examined. Upon parkin over-expression and mitochondrial depolarization with CCCP for 4 h, rapid degradation was observed only for the proteasome substrate MFN1 (Figure S1G). In contrast, degradation of OMM and inner- mitochondrial proteins was severely compromised in pentaKO cells compared to controls (Figure S1G-I), indicating that the degradation of the aforementioned OMM and IMM proteins in parkin-HeLa cells is mostly mediated by autophagy.

***Inner mitochondrial material is targeted to separate, late degradation pathways***

Although inner mitochondrial markers co-localized with TOM20 4 h after mitophagy induction (Figure 1B), the remaining TOM20 puncta that appeared after 14 h (likely representing OMM autophagolysosomes, see above) were clearly separate from inner mitochondrial material (Figure 1B). Moreover, most of the SSBP1 and CS-positive inner mitochondrial staining patterns at this late time point appeared as large mitochondrial aggregates of fragmented mitochondria rather than puncta (Figure 1B). Similar structures showing TOM20-positive mitochondrial condensates upon activation of PINK1/parkin-dependent mitophagy have been described previously as mitochondrial-aggresomes or mitochondrial aggregates [26,27]. While the presence of TOM20-negative mitochondrial condensates is surprising and has not been specifically described before, the morphology of these structures is similar to the ones described by Lee *et al.* [27]. Thus, mitochondrial condensates will be referred here as mitochondrial-aggresomes or mitochondrial aggregates.

To quantify the degradation dynamics of each mitochondrial sub-compartment, CellProfiler image-based analysis was performed at mid- and late stages of mitophagy (4 h vs. 14 h CCCP treatment) (Figure S2A-F). The mean number as well as the size of TOM20-positive (OMM) mitochondrial-aggresomes present after 4 h CCCP drastically decreased after 14 h of CCCP treatment (Figure S2A and B). Instead, TOM20 shapes were converted to (autophagolysosomal) puncta that were significantly degraded over the time course of mitophagy (Figure 1B, F and Figure S1E-F) unless when blocked by BafA (Figure 1H and I). On the other hand, the mean number of COX IV-positive (IMM) mitochondrial material only decreased to half at late stages of mitophagy, similar to SSBP1-positive mitochondrial-aggresomes (Figure S2A and B). The size of IMM or matrix aggresomes remained similar or only decreased slightly, respectively (Figure S2C and D). The decrease observed on IMM clump number can be explained by an increased appearance of IMM puncta at later stages of mitophagy, while matrix puncta were barely detectable and did not seem to change up to the 14 h time point (Figure S2E). Interestingly, the mean number of observed IMM puncta at late stages of mitophagy (14 h) was similar to the ones of OMM after 4 h of mitochondria depolarization, suggesting a similar -but delayed- autophagic delivery of IMM proteins (Figure S2E).

In order to confirm if the remaining inner- mitochondrial-aggresomes were eventually targeted by the autophagy machinery, a delayed autophagy-inhibiting experiment was performed. Here, mitochondrial depolarization was extended to 24 h and BafA was administered 8 h after the initiation of mitophagy (Figure 2A and B), allowing the formation of TOM20-negative mitochondrial-aggresomes but subsequently blocking the delivery of autophagosomes to the lysosome network. Interestingly, a significant amount of TOM20- and SSBP1-positive puncta were observed after delayed autophagy inhibition, when comparing to extended depolarizing conditions alone (Figure 2A and B). These data suggest lysosome-dependent steps for the later processing of TOM20-negative mitochondrial aggresomes and additionally validate lysosomal-dependent degradation of TOM20 puncta. Thus, there could be at least two distinct pools of mitochondrial proteins targeted via autophagy; the targeting of OMM proteins would occur first followed by a later autophagy targeting of inner- mitochondrial proteins (≥ 8 h CCCP).

As shown above, TOM20-negative mitochondrial aggresomes were positive for ubiquitin and targeted by the autophagy adaptors p62 and TAX1BP1 (Figure 1C-E). In addition, similar to TOM20-positive puncta, both endogenous as well as overexpressed forms of LC3B either overlapped or partially co-localized with COX IV puncta (Figure 2C and Figure S1E). Moreover, COX IV puncta merged with LAMP1-positive lysosomes at later time points (14 h CCCP) (Figure 2E) and co-localized with LysoTracker (Figure 2D). Similar to what was observed for the mitochondrial aggresome size, the relative cell area of OMM, IMM or matrix puncta was reduced when comparing early to late mitochondrial depolarization times (Figure S2F), even though the relative area of IMM puncta seemed to be larger than the ones of OMM or matrix puncta (Figure S2F). Remarkably, the matrix components remained without any LAMP1 co-localization up to 14 h CCCP treatment (Figure 2F). Taken together, these data indicate that inner mitochondrial material is targeted to the autophagy machinery and delivered to the lysosome degrading network at a later stage of the pathway (8-14 h CCCP), suggesting that PINK1/parkin-dependent mitophagy can trigger mitochondrial turnover in a sub-compartment specific manner and not only by promoting the engulfment of entire mitochondria.

In order to confirm piecemeal mitophagy in cell lines with endogenous parkin levels, HEK293 cells were exposed to extended mitochondria depolarization and the presence of TOM20-positive puncta that were negative for COX IV, SSBP1 or CS was observed (Figure S2H). Importantly, these events could also be validated in human NPCs (Figure S2I). Although the kinetics of mitophagy were much slower than in parkin-overexpressing cell clones, these findings suggest that distinct stepwise mitochondrial sub-compartment degradation can also take place in cells with endogenous parkin expression levels.

To gain higher resolution insight into the process of mitophagy in these cells, transmission electron microscopy (TEM) imaging was performed. Consistently, elongated mitochondria were observed under basal conditions (Figure S3A). However, upon mitochondrial depolarization, mitochondrial-aggregates as well as mitochondria being engulfed by autophagosomes were observed (Figure S3B). In addition, examples of engulfed mitochondria with damaged OMM could be observed (Figure S3C). These findings confirm autophagy-dependent mitochondrial turnover in WT-parkin HeLa cells and indicate that OMM rupture might occur during mitophagy.

***Mitophagy is regulated by several distinct proteasome-dependent steps***

There is a number of immediate-early proteasome targets that are removed from the OMM, which play an important role in the initiation of mitophagy [21,28,29]. Indeed, pretreatment with the proteasome inhibitors MG132 (Figure S1A) or Bortezomib (Figure S1B) not only reduced the degradation of the known proteasomal-dependent targets MFN1 and TOM70, respectively, but also compromised the degradation of all mitochondrial sub-compartment markers after both intermediate and long depolarization times. Indeed, when the proteasome was inhibited throughout the time course of mitophagy, all mitochondrial sub-compartment markers remained colocalized for at least 18 h, both in parkin-overexpressing HeLa cells and in HEK293 cells expressing endogenous levels of parkin (Figure S2J and K).

Next, we wondered if analogous to the proteasome dependence for mitophagy initiation at the OMM (see above), progression of mitophagy through the IMM could also be influenced by proteasome activity. Thus, a delayed-proteasome inhibition experiment was designed where proteasome inhibition was achieved by MG132 treatment together with mitochondrial depolarization with CCCP at several time points (Figure 3A). As expected, when mitochondria depolarization was extended up to 24 h, inner mitochondrial proteins were almost completely degraded (Figure 3B). Interestingly, when the proteasome was inhibited after TOM20 removal had taken place (24 h CCCP + 8 h MG132), the degradation of inner mitochondrial proteins was reduced (Figure 3B). Quantification of Western blot band densities revealed a trend of increased IMM protein COX IV and the matrix protein SSBP1 upon delayed inhibition of the proteasome, while a significant increase was found for CS under the same conditions in comparison to CCCP treatment only (Figure 3C).

To visualize proteasome influence on the late stages of mitophagy, morphology of mitochondrial remnant entities was analyzed in a quantitative manner, comparing extended CCCP treatment only (24 h CCCP) to extended CCCP treatment combined with late proteasome inhibition (24 h CCCP + 8 h MG132) (Figure 3D-F). Under normal depolarization conditions, the OMM was reduced to very few TOM20-postive puncta and SSBP1 appeared to be fully degraded. Surprisingly, once MG132 was added at an intermediate mitophagy stage (>8 h CCCP), the OMM seemed to still be reduced to TOM20-positive puncta but the matrix sub-compartment remained and appeared in SSBP1-matrix positive aggregates (Figure 3D). Single cell analysis of the average SSBP1-positive aggregate number and size showed a significant increase of both parameters when proteasome inhibitor was added after 8 h of CCCP treatment (Figure 3E and F). Thus, proteasome action may be involved in the degradation of inner- mitochondrial remnants, adding a possible novel step for the progression of mitophagy.

***Global proteome measurements confirm proteasome-sensitive steps in mitophagy***

To extend these findings to the proteomic level, we performed quantitative mass spectrometry measurements of the proteasome inhibition experiment using whole cell lysate samples in several experimental settings (Figure 4A). All three replicates of each experimental set-up showed high overlap in protein identification and high correlation in protein regulation (Figure S4A-E). Stepwise degradation of mitochondrial sub-compartments was in accordance with previous studies [20]. Indeed, while OMM and IMS proteins were mostly degraded within 8 h of CCCP treatment (Figure S4F), IMM and especially matrix protein degradation only took place at later stages of mitophagy (24 h CCCP) (Figure S4G).

Effects of proteasome inhibition prior to mitochondria depolarization (t -0.5 h) was analyzed on parkin-HeLa cells undergoing mitophagy. In agreement with previous results, CCCP failed to induce mitophagy after pretreatment with MG132. Compared to cells without MG132 treatment, OMM and IMS proteins primarily affected during the first 8 h of mitophagy were significantly less degraded after pretreatment with the proteasomal inhibitor MG132 (Figure 4B).

Next, the involvement of proteasome activity during late stages of mitophagy was assessed. In samples where proteasomal activity was inhibited after 8 h of CCCP treatment (24 h CCCP, t +8 h MG132), the degradation of OMM and IMS proteins was largely unaffected (Figure 4C). However, the degradation of IMM and matrix proteins was strongly reduced (Figure 4C, lower panel). In the presence of MG132 after 8 h of CCCP treatment most of the regulated proteins (p≤0.05; indicated by the 5% curves; p≤0.1; indicated by the 10% curves) are annotated for these sub-mitochondrial localizations (Figure 4C, upper panel) (Supplementary Table 1). For example, the cluster of mitochondrial ribosomal proteins of the large subunit (MRPL) was significantly (p≤0.05) less degraded after delayed addition of the proteasome (Figure 4C), underscoring that mitophagy of the matrix depends on a second, delayed proteasome-regulated step.

**Discussion**

PINK1/parkin-dependent mitophagy removes damaged mitochondria in stressed cells via autophagy [18]. Wholesale mitophagy might be the result of this pathway, but sequential mitochondrial sub-compartment turnover may also play a role [22,26,30,31]. Here we report parkin-dependent piecemeal mitophagy involving step-by-step sub-compartment degradation mechanisms where both the ubiquitin proteasome system (UPS) and autophagy pathways appear to be involved.

***Distinct subsets of OMM proteins are subject to immediate-early proteasomal and delayed autophagic degradation***

Proteasome degradation is known to be involved in early stages of PINK1/parkin-dependent mitophagy by contributing to mitochondrial fission in order to facilitate mitochondrial engulfment by autophagosomes [21,32]. This is likely due to the proteasome-dependent removal of the mitochondrial fusion factor MFN1. In addition, previous studies involving the inhibition of proteasome degradation by either MG132 or lactacystin have shown that PINK1/parkin mitophagy was prevented under proteasome inhibition conditions [21,28]. Our data support the notion that proteasome-degradation is essential for successful mitochondria elimination and indicate that early elimination of OMM substrates (i.e: MFN1) can be considered a first checkpoint for mitophagy initiation.

In addition to proteasomal turnover, the extended ubiquitination events on the OMM promote the recognition of autophagy adaptors [5,16,33] . Indeed, ubiquitination events of OMM proteins and TOM20-positive mitochondrial-aggresomes triggered the recognition of autophagy adaptors in our model (Figure 1C-E). Those events were followed by the formation of autophagosomes, as evidenced by TOM20 puncta positive for LC3B (Figure 1F and Figure S1E). However, not all TOM20 puncta observed were co-localizing with L3CB structures. This could reflect (1) the involvement of additional mammalian ATG8 family members [34] in the removal of TOM20 or (2) LC3B-independent pathways on the removal of OMM substrates. Indeed, other forms of OMM turnover that do not require autophagosome formation for ultimate delivery to lysosomes have been previously described and are known as MDVs [22]. While the formation mechanisms of MDVs are not yet fully understood, it has been suggested that MDVs serve as a first line of defense against mild mitochondrial stress conditions [31,35]. Thus, OMM turnover could occur via direct lysosomal delivery of specific OMM substrates as well as proteasome/autophagy dependent pathways, the latter being more prominent under strong mitophagy inducing conditions, as studied here.

***Inner mitochondrial material is targeted to separate, late degradation pathways***

The turnover rate of inner- mitochondrial-aggresomes appeared much distinct from that of OMM proteins (Figure 1B). However, delayed autophagy inhibition experiments suggested an involvement of the autophagy machinery for the turnover of inner- mitochondrial-aggresomes (Figure 2A and B). Indeed, similarly to what was observed for TOM20 puncta turnover, the involvement of autophagic degradation in the turnover of inner- mitochondrial aggresomes could be confirmed by co-localization of COX IV puncta with LC3B as well as LysoTracker (Figure 2C and D).

Overall, these data point to a temporal selectivity of the autophagic machinery, leading to the involvement of two separate autophagic waves. However, while further studies are needed to confirm a possible autophagy adaptor selectivity between the recognition of ubiquitinated OMM or inner- mitochondrial substrates, prohibitin 2 has been proposed to act as an inner- mitochondrial adaptor protein that directly interacts with LC3B [36]. The presence of other specific IMM proteins capable of interacting with autophagosomes could offer future insights into the upstream events that trigger selective autophagic removal of different mitochondrial sub-compartments.

The exact nature of TOM20-negative inner mitochondrial-aggregates remains to be further characterized, particularly with regard to the OMM composition at advanced stages of mitophagy. TEM showed examples of OMM-damaged mitochondria engaged with autophagosomes after 4 h CCCP treatment (Figure S3C). These data are in agreement with other studies reporting OMM-damage upon PINK1/parkin mitophagy induction [28,36]. Further studies shall elucidate the specific temporal dynamics and mechanisms of the interactions of mitochondrial sub-compartments with their respective degradation machineries.

Moreover, while our model of parkin-dependent mitophagy globally differs from a wholesale mitophagy turnover model, the sporadic presence of OMM and matrix material engaged in LC3B structures (Figure 1F and Figure S1E) and the presence of TOM20 puncta negative for LC3B, suggests that parkin-dependent mitochondrial turnover might combine both wholesale and piecemeal mitophagy turnover pathways. Thus, while most of the evidence on endogenous parkin-dependent wholesale mitophagy has been reported on tissue-specific cells where mitochondrial survival is crucial for their metabolic or physiological demands (i.e: neurons and hepatocytes) [8,37,38], it is possible that when cell survival is not strictly dependent on mitochondrial functions, wholesale mitophagy partially switches to piecemeal mitophagy; possibly involving MDV formation as well selective autophagic removal of mitochondrial sub-compartment proteins (Figure 1 and 2).

In addition, the kinetics of the pathway might play an important role on sub-compartment availability since mitophagy progression has been shown to differ between cell lines, even under parkin-overexpression conditions [39–41]. Actually, while endogenous parkin-dependent mitophagy in HEK293 and NPC cells revealed TOM20 staining patterns clearly distinguishable from inner- mitochondrial material, the mitophagy process was more protracted in cell lines expressing lower levels of endogenous parkin (Figure S2H-J).

***Mitophagy is regulated by several distinct proteasome-dependent steps***

Mitophagy initiation involves parkin-dependent ubiquitination of specific OMM proteins that are directly targeted to the proteasome, especially affecting proteins that are involved in mitochondrial fusion or transport [1,21,28,42]. As expected, proteasome inhibition prevented the elimination of proteasomal-dependent substrates (Figure S1A and B) and compromised the overall mitochondrial turnover in different cell lines (Figure S2J and K), indicating that OMM-proteasome degradation is upstream of autophagy activation during parkin-dependent mitophagy. These data support the notion that a first proteasomal-dependent targeting of the OMM is needed for sub-sequent mitophagy progression [28].

On the other hand, advanced stages of parkin-dependent mitophagy were characterized by the presence of inner- mitochondrial aggresomes (Figure 1B, 14 h CCCP). Such structures showed to be ubiquitinated and recognized by the autophagy adaptors p62 and TAX1BP1 (Figure 1C-E), similarly to what we observed for TOM20-positive mitochondrial aggresomes (Figure 1B, 4 h CCCP).

Because OMM turnover involved both proteasome and autophagy-dependent pathways, we hypothesized that a similar contribution of the two pathways could influence the degradation of inner- mitochondrial aggresomes. Surprisingly, delayed proteasome inhibition promoted an increase in the amount of inner- mitochondrial aggresomes after extended mitochondrial depolarization (Figure 3D), suggesting proteasome-dependent targeting of inner- mitochondrial aggresomes. Importantly, delayed proteasome inhibition did not only affect the turnover of matrix proteins alone but also had an influence on COX IV turnover (Figure 3A), indicating that the observed TOM20-negative mitochondrial aggresomes are indeed COX IV-positive. In addition, because COX IV turnover seems to be autophagy-dependent (Figure 2C) and the initial OMM-turnover seems to be bound to a prior proteasomal-dependent OMM-degradation, it is possible that the delayed proteasomal inhibition indirectly impairs the autophagic machinery to target IMM-positive mitochondrial aggresomes.

While the exact mechanisms explaining how the UPS may interact with inner- mitochondrial aggresomes remains to be elucidated, it is possible that late OMM-turnover promotes a partial exposure of the IMM to the cytosol, allowing the UPS to act upon IMM substrates. Indeed, Wei and colleagues reported a clear OMM-rupture that allowed phagophore accessibility to the IMM in a prohibitin-dependent manner [36], thus we suggest that OMM-ruptured patches could facilitate access of proteasomes to IMM exposed regions. Alternatively, proteasomes might remove newly synthesized nuclear-encoded inner mitochondrial proteins, adding to the natural turnover of such proteins.

**Conclusions**

Taken together, our findings demonstrate that PINK1/parkin mitophagy occurs in a sequential manner, where outer mitochondrial membrane autophagy cargo is firstly separated from inner mitochondrial material for sub-sequent autophagolysosomal targeting. Importantly, such piecemeal mitophagy might also be observed under endogenous parkin expressing conditions, even though following a considerably slower kinetics. Additionally, we provide evidence of a potential proteasome-sensitive step which may regulate the progression of mitophagy towards complete mitochondria degradation. In sum, our study reveals later additional mitophagy steps: involving the delivery of mitochondrial sub-compartments to the lysosomal machinery in two separate consecutive autophagy waves and additionally suggesting the involvement of proteasomal action at later stages of mitophagy, similarly as it is observed at early stages of PINK1/parkin mitophagy on OMM substrates (Figure 5).

**Materials and Methods**

**Cell culture, treatments and transfection**

HeLa cells stably expressing 3xFlag-parkin WT [43] as well as HEK293 cells were cultured in high-glucose Dulbecco’s modified Eagle’s medium (DMEM) supplemented with 10% (v/v) fetal bovine serum (FBS) at 37°C with 5% CO_2_. PentaKO HeLa cells were a kind gift from Prof. Richard Youle (National Institute of Health, Bethesda, USA) and were used as described [16]. Depolarization of mitochondria was achieved by adding 10 μM CCCP (Sigma, C2759) to the media. For better visualization of LC3B puncta, transfection of pEGFP-C1-LC3B [46] in WT-parkin HeLa cells was done. Briefly, 24 h prior to CCCP treatment, cells were transfected with pEGFP-C1-LC3B using FuGene6® (Promega, E269A) according to manufacturer instructions.

For proteasomal inhibition studies, cells were pretreated with 20 μM MG132 (Sigma, C2211) for 30 min before addition of an equal volume of media containing 20 μM CCCP, bringing the final concentrations of both MG132 and CCCP to 10 μM for the indicated incubation times. In delayed proteasome inhibition experiments cells were treated with either 10 μM MG132 or 400 nM Bortezomib (Sellekchem, S1013) for the indicated time points before or after CCCP administration. For autophagy inhibition experiments, cells were treated with 400 nM BafA (Sigma, 1793) for the indicated time points before or after CCCP administration. All treatments were combined with 10 μM CCCP.

NPCs were derived from healthy, human induced pluripotent stem cells and have been previously characterized and labelled in this manuscript as wtNPC line 1 [44] or wtNPC line 2 [45]. NPCs were cultured on Matrigel (Corning, 354320) coated plates in NPC medium containing a 1:1 mixture of DMEM-Ham’s F12 (Merck, F4815) with Neurobasal (Gibco, 21103049) containing L-glutamine (Gibco, 35050061) and Penicillin/Streptomycin (Merck, A2213). Media was supplemented with N2 (Gibco, 17502-048) and B27 (Gibco, 12587-010) plus 3 µM CHIR 99021 (Axon Medchem, 1386), 200 µM ascorbic acid (Sigma-Aldrich, 50-81-7) and 0.5 µM purmorphamine (Calbiochem, 540220). Mitochondria depolarization was achieved by adding 100 μM Antimycin A supplemented with 10 µM Y-27632 (Selleckchem, S1049) for the indicated time points.

**Antibodies**

**Table 1**: List of primary and secondary antibodies and concentration used in this study

| Primary antibodies | | |
| --- | --- | --- |
| TOM70 | 1:1000 | ProteinTech, 14528-1-AP |
| TOM20 | 1:1000 (IF) | Santa Cruz, sc-11415 |
| TOM20 | 1:6000 (WB)  1:1000 (IF) | Proteintech, 11802-1-AP |
| VDAC | 1:30000 | Millipore, AB10527 |
| Total ubiquitin (FK2 clone) | 1:1000 | Enzo Life Sciences, BML-PW88100100 |
| p62/SQSTM | 1:1000 | BD Biosciences, 610832 |
| COX IV | 1:6000 (WB)  1:1000 (IF) | Cell Signaling, 4850 |
| CS | 1:6000 (WB) 1:1000 (IF) | GeneTex, GTX110624 |
| SSBP1 | 1:500 (WB)  1:1000 (IF) | R&D Systems, AF6588 |
| Vinculin | 1:6000 | Sigma Aldrich, V9131 |
| MFN1 | 1:10000 (WB)  1:1000 (IF) | Abnova, H00055669-M04 |
| β-actin | 1:5000 | Sigma Aldrich, A5441 |
| LAMP1 | 1:500 | DSHB Hybridoma Bank, clone H4A3 |
| LC3B | 1:250 | GeneTex, GT3612 |
| TAX1BP1 | 1:500 | Novus Biologicals, NBP1-86662 |
| Parkin | 1:10000 (WB)  1:1000 (IF) | Cell Signaling, 4211S |
| Secondary antibodies | | |
| Goat anti-mouse HRP | 1:10000 | Amersham Pharmacia, 115-035-003 |
| Goat anti-rabbit HRP | 1:10000 | Amersham Pharmacia, 111-035-003 |
| Goat anti-sheep HRP | 1:10000 | Amersham Pharmacia, 718-035-147 |
| Alexa Fluor 647 donkey anti-sheep | 1:1000 | Invitrogen, A-21448 |
| Alexa Fluor 488 donkey anti-mouse | 1:1000 | Invitrogen, A-21202 |
| Alexa Fluor 488 donkey anti-rabbit | 1:1000 | Invitrogen, A-21206 |
| Alexa Fluor 568 donkey anti-mouse | 1:1000 | Invitrogen, A-10037 |
| Alexa Fluor 568 donkey anti-rabbit | 1:1000 | Invitrogen, A-10042 |
| Alexa Fluor 350 goat anti-rabbit | 1:1000 | Invitrogen, A-11046 |
| Alexa Fluor 350 goat anti-mouse | 1:1000 | Invitrogen, A-10045 |

**Western blotting**

Cell harvest was performed directly after a single wash with phosphate-buffered saline (PBS). Cells were pelleted at 972 x *g* for 1 min at 4°C. Pellets were resuspended in urea lysis buffer (10 mM Tris, pH 8.0, 100 mM NaH_2_PO_4_, and 8 M urea) and passed through a 26-gauge needle for at least 5 times. Debris was pelleted at 3151 x *g* for 15 min at 4°C to obtain whole cell lysate supernatants. Total protein was measured using the standard Bradford Protein Assay Kit (Bio-Rad). Samples were separated on 10% or 15% polyacrylamide gels or 4-20% gradient gels (TruPAGE precast gels, Merck) and run for 2 h at 140 V or 1 h at 100 V, respectively. Proteins were transferred to polyvinylidene fluoride membranes (Immobilon-P, Merck) for 2 h at 100 V, blocked with 5% bovine serum albumin or non-fat dry milk in Tris-buffered saline buffer containing 0.1% Tween-20 (TBS-T). Membranes were incubated overnight with the indicated primary antibodies, washed at least three times with TBS-T and further incubated with matching horseradish peroxidase conjugated secondary antibodies diluted in 5% milk. Chemiluminescent reaction was carried out with Western blot chemiluminescent-horseradish peroxidase substrate (Millipore). Three TBS-T washings of at least 5 min were performed between each incubation step. Ultracruz autoradiography films (Santa Cruz Biotechnology) were used for band visualizations.

**Immunostaining and microscopy**

Cells were seeded on pre-coated coverslips with poly-D-lysine (Sigma, P6407) and treated for the indicated time points. In addition, NPCs were cultured on Matrigel coated plates in NPC medium as previously described. Cells were fixed with 4% paraformaldehyde in PBS for 20 min at room temperature (RT). Cell permeabilization was achieved with 1% Triton X-100 for 5 min at RT and cells were washed three times with PBS. Blocking was performed with 10% FBS in PBS for 1 h at RT, followed by primary antibody incubation with 5% FBS for 2 h at RT. Cells were washed three times with PBS before incubating for 1 h at RT with the secondary antibody diluted in PBS with 10% FBS. Nuclei were stained with 2 μg/ml Hoechst (Molecular Probes, 3570) after washing the cells for at least three times with PBS. Cells were mounted onto coverslips using Fluorescent Mounting Medium (Dako). Imaging was performed using an AxioImager microscope equipped with an ApoTome imaging system using 63X or 100X objectives (Carl Zeiss). The images were processed with AxioVision 4.9.1 software (Carl Zeiss). For immunostaining of endogenous LC3B, normal goat serum instead of FBS was used.

For lysotracker staining of fixed cells, 500 nM LysoTracker RED (Invitrogen, LT528) was added to the cell culture 30 min before fixation. Then cells were washed with ice-cold DMEM for 10 min followed by two gentle washes of ice-cold PBS. Cells were fixed with 4% paraformaldehyde and the immunostaining was performed as previously described. LysoTracker red was imaged on 568 channel and mitochondrial markers were stained with Alexa fluor 350 and imaged on the Hoechst channel with an Apotome imaging system as described above.

**CellProfiler analysis**

Image analysis of mitochondrial morphology was performed using CellProfiler (4.2.1 version). The identification of mitochondrial-aggresomes and puncta was performed as follows: all mitochondrial objects in between a range of 6-500 px range were identified and merged together as a new single object if the distance between the initial primary objects was ≤2 px apart. New merged single objects with a minimal area of 300-500 px were considered aggresomes and objects with an area <300 px were considered dots.

For delayed inhibition experiments, mitochondrial objects of at least 30 px were identified and merged as a single object if ≤15 px apart. New merged single objects with an area below 300 px were considered puncta, objects with an area of >300 were considered aggresomes.

**Transmission electron microscopy**

For TEM analysis, HeLa cells were cultured and treated with CCCP as described above for IFM, except cells were grown on 22 x 22 mm square glass coverslips. Cells were prefixed in culture medium with 2.5% glutaraldehyde and 4% paraformaldehyde, pH adjusted to 7.2, for 20 minutes at 37°C. Cells were then fixed in 0.1 M HEPES, 4 mM CaCl_2_, 2.5% glutaraldehyde (Serva Electrophoresis,), 4% formaldehyde (Science Services), pH 7.2 for 1 h at RT plus 3 h at 4°C, the fixative being replaced a few times. Cells were postfixed with 1% OsO_4_ (in distilled water) for 60 min at 4°C and then incubated in 1% uranyl acetate overnight at 4°C. Between each step the samples were washed 3-4 times for 5 min each with distilled water. Dehydration of the samples in ethanol, infiltration with Epon (Serva Electrophoresis) and flat embedding was done following standard procedures [50]. Ultrathin sections (~60-70 nm) were cut with a diamond knife (type ultra 35°; Diatome, Biel, Switzerland) with an EM UC6 ultramicrotome (Leica, Wetzlar, Germany) and mounted on single-slot Pioloform-coated copper grids (Plano, Wetzlar, Germany). Sections were stained with uranyl acetate and lead citrate [51] and viewed with a JEM-2100 transmission electron microscope (JEOL, Tokyo, Japan) operated at 80 kV. Micrographs were taken using a 4000 x 4000 charge-coupled device camera (UltraScan 4000; Gatan, Pleasanton, CA) and Gatan Digital Micrograph software (version 1.70.16.). Image brightness and contrast were adjusted and figures assembled using Adobe Photoshop 8.0.1 and Inkscape 1.0 beta.

**Quantitative proteomics**

For all proteomics studies, 10 µg of protein lysate per dimethyl labeling channel were used. First, disulfide bonds were reduced with 10 mM dithiothreitol for 1 h at RT, followed by alkylation with 55 mM iodoacetamide for 1 h at RT in the dark. Prior to overnight digestion with trypsin (Promega Corporation, V5280), pre-digestion with lysyl endopeptidase (Wako Chemicals, 121-05063) for 3 h was performed in a peptidase-protein ratio of 1:100 at RT. Digestion was quenched by adding 1% trifluoroacetic acid. Peptides were dimethyl-labeled on C18 StageTips as described previously [47]. Label efficiency and peptide mixing ration of 1:1:1 was checked in pilot LC-MS/MS runs. For peptide fractionation the Pierce™ High pH Reversed-Phase Peptide Fractionation Kit (Thermo Fisher Scientific, 84868) was applied as described earlier [20]. Prior to LC-MS/MS measurement, peptides were desalted on C18 StageTips. All fractions were analyzed on an Q Exactive HF mass spectrometer (Thermo Fischer Scientific), online-coupled to Easy-nLC 1200 UHPLC (Thermo Fischer Scientific) as described earlier [20]. In short, peptides were separated on a 20 cm analytical column (75 µm ID PicoTip fused silica emitter, New Objective) in-house packed with ReproSil-Pur C18-AQ 1.9 μm resin (Dr Maisch GmbH), by a fraction-specific segmented 90 min gradient of solvent A (0.1% formic acid) and solvent B (0.1% formic acid in 80% acetonitrile) at 40°C and a 200 nl/min flow rate. Electrospray ionization was used to ionize eluted peptides. The mass spectrometer was operated in a positive ion mode. Full MS spectra were recorded in a scan range of 300-1650 m/z at resolution 60k. The top 12 most abundant multiple charged ions were selected for HCD fragmentation (AGC target: 3e6, Maximum IT: 25 ms). MS2 spectra were acquired at resolution 30k (AGC target: 1e5, Maximum IT: 45 ms).

LC-MS/MS raw data were searched against the Uniprot Homo sapiens database (released 11.12.2019, 96,818 entries) and commonly observed contaminants using the Andromeda search engine integrated into the MaxQuant software suite (version 2.0.3.0) [48]. All search parameters were kept to default except for the following. Carbamidomethylation of cysteine in addition to oxidation of methionine and protein N-terminal acetylation were set as fixed and variable modifications, respectively. Trypsin was selected as a protease and a maximum of two missed cleavages were allowed. Light (28.03 Da), intermediate (32.06 Da), and heavy (36.08 Da) dimethyl labels were specified for N termini and lysine residues. A minimum of two peptide ratio counts were required for protein quantification. Precursor ion mass tolerance was set to 4.5 ppm and fragment ions to 20 ppm, re-quantify and match-between runs between fractions of the same sample were enabled. The mass spectrometry proteomics data have been deposited to the ProteomeXchange Consortium via the PRIDE [49] partner repository with the dataset identifier PXD034136.

**Statistics**

CellProfiler analysis of mitochondrial sub-compartment degradation as well as late inhibition experiments was performed using one-way ANOVA and Tukey test as post-hoc analysis test, using Graph Pad prism 6 software. Graphs show mean ± SEM of cell populations from at least three independent experiments. Significance is indicated by asterisks as described in figure legends.

Statistical analysis of quantitative proteomics data was performed with Perseus software suite (version 1.6.15.0) [52]. First, all reverse and potentially contaminant hits were filtered out. For protein annotation MitoCarta 3.0 was used as a resource [53]. Only protein ratios present in two out of three replicates were considered for downstream analysis. To identify significantly regulated proteins between different treatments, a one-sample t-test was performed (p-value ≤ either 0.1 or 0.05).

Venn Diagrams, representing the overlap of protein identification between replicates were performed with the online tool <http://www.interactivenn.net/> (accessed on 14^th^ April 2022) [54].

**Acknowledgements**

This work was funded by the German Research Foundation (DFG) Research Training Group GRK2364 and the Hertie Foundation. We thank David Schüssele and Tassula Proikas-Cezanne for helpful suggestions and critically reading the manuscript. We also thank Richard Youle and Chunxin Wang for sharing the PentaKO HeLa cell line (also acknowledging Henrietta Lacks and her family).

**References**

1. Terradas AL, Zittlau KI, Macek B, Fraiberg M, Elazar Z, Kahle PJ. Regulation of mitochondrial cargo-selective autophagy by posttranslational modifications. J Biol Chem 2021 ; 297(5):101339. Pubmed: PMID 34688664

2. Kitada T, Aakawa S, Hattori N, Matsumine H, Yokochi M, Mizuno Y, Shimizu N. Mutations in the parkin gene causeautosomal recessive juvenile parkinsonism. Nat Lett 1998 ; 169(1993):166–9.

3. Valente EM, Abou-Sleiman PM, Caputo V, Muqit MMK, Harvey K, Gispert S, Ali Z, Del Turco D, Bentivoglio AR, Healy DG, et al. Hereditary early-onset Parkinson’s disease caused by mutations in PINK1. Science (80- ) 2004 ; 304(5674):1158–60. Pubmed: PMID 15087508

4. Springer W, Kahle PJ. Regulation of PINK1-Parkin-mediated mitophagy. Autophagy 2011 ; 7(3):266–78. Pubmed: PMID 21187721

5. Geisler S, Holmström KM, Skujat D, Fiesel FC, Rothfuss OC, Kahle PJ, Springer W. PINK1/Parkin-mediated mitophagy is dependent on VDAC1 and p62/SQSTM1. Nat Cell Biol 2010 ; 12(2):119–31. Pubmed: PMID 20098416

6. Georgakopoulos ND, Wells G, Campanella M. The pharmacological regulation of cellular mitophagy. Nat Chem Biol 2017 ; 13(2):136–46. Pubmed: PMID 28103219

7. Kasianowicz J, Benz R, McLaughlin S. The kinetic mechanism by which CCCP (carbonyl cyanide m-chlorophenylhydrazone) transports protons across membranes. J Membr Biol 1984 ; 82(2):179–90.

8. Ashrafi G, Schlehe JS, LaVoie MJ, Schwarz TL. Mitophagy of damaged mitochondria occurs locally in distal neuronal axons and requires PINK1 and Parkin. J Cell Biol 2014 ; 206(5):655–70. Pubmed: PMID 25154397

9. Chung-Han H, Atossa S, Ashley E. G, Alexandre B da C, Lena F. B, Lawrence ES, Birgitt S, Krainc D, D T, Palmer1, 2 and XW. Functional Impairment in Miro Degradation and Mitophagy Is a Shared Feature in Familial and Sporadic Parkinson’s Disease. Physiol Behav 2017 ; 176(5):139–48.

10. Sekine S. PINK1 import regulation at a crossroad of mitochondrial fate: The molecular mechanisms of PINK1 import. J Biochem 2020 ; 167(3):217–24. Pubmed: PMID 31504668

11. Kondapalli C, Kazlauskaite A, Zhang N, Woodroof HI, Campbell DG, Gourlay R, Burchell L, Walden H, MacArtney TJ, Deak M, et al. PINK1 is activated by mitochondrial membrane potential depolarization and stimulates Parkin E3 ligase activity by phosphorylating Serine 65. Open Biol 2012 ; 2(MAY). Pubmed: PMID 22724072

12. Koyano F, Okatsu K, Kosako H, Tamura Y, Go E, Kimura M, Kimura Y, Tsuchiya H, Yoshihara H, Hirokawa T, et al. Ubiquitin is phosphorylated by PINK1 to activate parkin. Nature 2014 ; 510(7503):162–6. Pubmed: PMID 24784582

13. Matsuda N. Phospho-ubiquitin: Upending the PINK-Parkin-ubiquitin cascade. J Biochem 2016 ; 159(4):379–85. Pubmed: PMID 26839319

14. Ordureau A, Sarraf SA, Duda DM, Heo JM, Jedrychowski MP, Sviderskiy VO, Olszewski JL, Koerber JT, Xie T, Beausoleil SA, et al. Quantitative proteomics reveal a feedforward mechanism for mitochondrial PARKIN translocation and ubiquitin chain synthesis. Mol Cell 2014 ; 56(3):360–75. Pubmed: PMID 25284222

15. Heo JM, Ordureau A, Paulo JA, Rinehart J, Harper JW. The PINK1-PARKIN Mitochondrial Ubiquitylation Pathway Drives a Program of OPTN/NDP52 Recruitment and TBK1 Activation to Promote Mitophagy. Mol Cell 2015 ; 60(1):7–20. Pubmed: PMID 26365381

16. Lazarou M, Sliter DA, Kane LA, Sarraf SA, Wang C, Burman JL, Sideris DP, Fogel AI, Youle RJ. The ubiquitin kinase PINK1 recruits autophagy receptors to induce mitophagy. Nature 2015 ; 524(7565):309–14. Pubmed: PMID 26266977

17. Dikic I, Elazar Z. Mechanism and medical implications of mammalian autophagy. Nat Rev Mol Cell Biol 2018 ; 19(6):349–64. Pubmed: PMID 29618831

18. Wade Harper J, Ordureau A, Heo JM. Building and decoding ubiquitin hains for mitophagy. Nat Rev Mol Cell Biol 2018 ; 19(2):93–108. Pubmed: PMID 29358684

19. Kolitsida P, Zhou J, Rackiewicz M, Nolic V, Dengjel J, Abeliovich H. Phosphorylation of mitochondrial matrix proteins regulates their selective mitophagic degradation. Proc Natl Acad Sci U S A 2019 ; 116(41):20517–27. Pubmed: PMID 31548421

20. Zittlau KI, Lechado-Terradas A, Nalpas N, Geisler S, Kahle PJ, MacEk B. Temporal Analysis of Protein Ubiquitylation and Phosphorylation During Parkin-Dependent Mitophagy. Mol Cell Proteomics 2022 ; 21(2):100191. Pubmed: PMID 34974192

21. Tanaka A, Cleland MM, Xu S, Narendra DP, Suen DF, Karbowski M, Youle RJ. Proteasome and p97 mediate mitophagy and degradation of mitofusins induced by Parkin. J Cell Biol 2010 ; 191(7):1367–80. Pubmed: PMID 21173115

22. Soubannier V, McLelland GL, Zunino R, Braschi E, Rippstein P, Fon EA, McBride HM. A vesicular transport pathway shuttles cargo from mitochondria to lysosomes. Curr Biol 2012 ; 22(2):135–41. Pubmed: PMID 22226745

23. Mindell JA. Lysosomal acidification mechanisms. Annu Rev Physiol 2012 ; 74:69–86. Pubmed: PMID 22335796

24. Klionsky DJ, Abdelmohsen K, Abe A, Abedin MJ, Abeliovich H, Arozena AA, Adachi H, Adams CM, Adams PD, Adeli K, et al. Guidelines for the use and interpretation of assays for monitoring autophagy (3rd edition). Autophagy 2016 ; 12(1):1–222. Pubmed: PMID 26799652

25. Zhou J, Tan SH, Nicolas V, Bauvy C, Yang N Di, Zhang J, Xue Y, Codogno P, Shen HM. Activation of lysosomal function in the course of autophagy via mTORC1 suppression and autophagosome-lysosome fusion. Cell Res 2013 ; 23(4):508–23. Pubmed: PMID 23337583

26. Hsieh CW, Yang WY. Omegasome-proximal PtdIns(4,5)P 2 couples F-actin mediated mitoaggregate disassembly with autophagosome formation during mitophagy. Nat Commun 2019 ; 10(1):1–12. Pubmed: PMID 30814505

27. Lee JY, Nagano Y, Taylor JP, Lim KL, Yao TP. Disease-causing mutations in Parkin impair mitochondrial ubiquitination, aggregation, and HDAC6-dependent mitophagy. J Cell Biol 2010 ; 189(4):671–9. Pubmed: PMID 20457763

28. Yoshii SR, Kishi C, Ishihara N, Mizushima N. Parkin mediates proteasome-dependent protein degradation and rupture of the outer mitochondrial membrane. J Biol Chem 2011 ; 286(22):19630–40. Pubmed: PMID 21454557

29. Wang H, Song P, Du L, Tian W, Yue W, Liu M, Li D, Wang B, Zhu Y, Cao C, et al. Parkin ubiquitinates Drp1 for proteasome-dependent degradation: Implication of dysregulated mitochondrial dynamics in Parkinson disease. J Biol Chem 2011 ; 286(13):11649–58. Pubmed: PMID 21292769

30. Yang JY, Yang WY. Bit-by-bit autophagic removal of parkin-labelled mitochondria. Nat Commun 2013 ; 4. Pubmed: PMID 24013556

31. McLelland GL, Soubannier V, Chen CX, McBride HM, Fon EA. Parkin and PINK1 function in a vesicular trafficking pathway regulating mitochondrial quality control. EMBO J 2014 ; 33(4):282–95. Pubmed: PMID 24446486

32. Twig G, Elorza A, Molina AJA, Mohamed H, Wikstrom JD, Walzer G, Stiles L, Haigh SE, Katz S, Las G, et al. Fission and selective fusion govern mitochondrial segregation and elimination by autophagy. EMBO J 2008 ; 27(2):433–46. Pubmed: PMID 18200046

33. Shi J, Fung G, Deng H, Zhang J, Fiesel F, Springer W, Li X, Luo H. NBR1 is dispensable for PARK2-mediated mitophagy regardless of the presence or absence of SQSTM1. Cell Death Dis 2015 ; 6(10):1–9. Pubmed: PMID 26512954

34. Johansen T, Lamark T. Selective Autophagy: ATG8 Family Proteins, LIR Motifs and Cargo Receptors. J Mol Biol 2020 ; 432(1):80–103. Pubmed: PMID 31310766

35. Sugiura A, McLelland G, Fon EA, McBride HM. A new pathway for mitochondrial quality control: mitochondrial‐derived vesicles. EMBO J 2014 ; 33(19):2142–56. Pubmed: PMID 25107473

36. Wei Y, Chiang W-C, Sumpter RJ, Mishra P, Levine B. Prohibitin 2 Is an Inner Mitochondrial Membrane Mitophagy Receptor. Cell 2018 ; 176(5):139–48.

37. Ylä-Anttila P, Vihinen H, Jokitalo E, Eskelinen EL. Chapter 10 Monitoring Autophagy by Electron Microscopy in Mammalian Cells. Methods Enzymol 2009 ; 451(C):143–64. Pubmed: PMID 19200881

38. Kim I, Lemasters JJ. Mitochondrial degradation by autophagy (mitophagy) in GFP-LC3 transgenic hepatocytes during nutrient deprivation. Am J Physiol - Cell Physiol 2011 ; 300(2):308–17. Pubmed: PMID 21106691

39. Kishi-Itakura C, Buss F. The use of correlative light-electron microscopy (CLEM) to study PINK1/Parkin-mediated mitophagy. Methods Mol Biol 2018 ; 1759(March 2017):29–39. Pubmed: PMID 28361485

40. Pickles S, Vigié P, Youle RJ. Mitophagy and Quality Control Mechanisms in Mitochondrial Maintenance. Curr Biol 2018 ; 28(4):R170–85. Pubmed: PMID 29462587

41. Grenier K, McLelland GL, Fon EA. Parkin- and PINK1-dependent mitophagy in neurons: Will the real pathway please stand up? Front Neurol 2013 ; 4 JUL(July):1–8.

42. Oshima Y, Cartier E, Boyman L, Verhoeven N, Polster BM, Huang W, Kane M, Jonathan Lederer W, Karbowski M. Parkin-independent mitophagy via drp1-mediated outer membrane severing and inner membrane ubiquitination. J Cell Biol 2021 ; 220(6). Pubmed: PMID 33851959

43. Geisler S, Jäger L, Golombek S, Nakanishi E, Hans F, Casadei N, Terradas AL, Linnemann C, Kahle PJ. Ubiquitin-specific protease USP36 knockdown impairs Parkin-dependent mitophagy via downregulation of Beclin-1-associated autophagy-related ATG14L. Exp Cell Res 2019 ; 384(2):111641. Pubmed: PMID 31550441

44. Bus C, Zizmare L, Feldkaemper M, Geisler S, Zarani M, Schaedler A, Klose F, Admard J, Mageean CJ, Arena G, et al. Human Dopaminergic Neurons Lacking PINK1 Exhibit Disrupted Dopamine Metabolism Related to Vitamin B6 Co-Factors. iScience 2020 ; 23(12). Pubmed: PMID 33299968

45. Schwarz L, Casadei N, Fitzgerald JC. Generation of R272Q, S156A and K572R RHOT1/Miro1 point mutations in iPSCs from a healthy individual using FACS-assisted CRISPR/Cas9 genome editing. Stem Cell Res 2021 ; 55:102469. Pubmed: PMID 34359002

46. Krebiehl G, Ruckerbauer S, Burbulla LF, Kieper N, Maurer B, Waak J, Wolburg H, Gizatullina Z, Gellerich FN, Woitalla D, et al. Reduced basal autophagy and impaired mitochondrial dynamics due to loss of Parkinson’s disease-associated protein DJ-1. PLoS One 2010 ; 5(2). Pubmed: PMID 20186336

47. Boersema PJ, Raijmakers R, Lemeer S, Mohammed S, Heck AJR. Multiplex peptide stable isotope dimethyl labeling for quantitative proteomics. Nat Protoc 2009 ; 4(4):484–94. Pubmed: PMID 19300442

48. Cox J, Mann M. MaxQuant enables high peptide identification rates, individualized p.p.b.-range mass accuracies and proteome-wide protein quantification. Nat Biotechnol 2008 ; 26(12):1367–72. Pubmed: PMID 19029910

49. Perez-Riverol Y, Bai J, Bandla C, García-Seisdedos D, Hewapathirana S, Kamatchinathan S, Kundu DJ, Prakash A, Frericks-Zipper A, Eisenacher M, et al. The PRIDE database resources in 2022: A hub for mass spectrometry-based proteomics evidences. Nucleic Acids Res 2022 ; 50(D1):D543–52. Pubmed: PMID 34723319

50. Rogowski M, Scholz D, Geimer S. Electron microscopy of flagella, primary cilia, and intraflagellar transport in flat-embedded cells. Methods Enzymol 2013 ; 524:243–63. Pubmed: PMID 23498744

51. Reynolds ES. The use of lead citrate at high pH as an electron-opaque stain in electron microscopy. J Cell Biol 1963 ; 17(1):208–12. Pubmed: PMID 13986422

52. Tyanova S, Temu T, Sinitcyn P, Carlson A, Hein MY, Geiger T, Mann M, Cox J. The Perseus computational platform for comprehensive analysis of (prote)omics data. Nat Methods 2016 ; 13(9):731–40. Pubmed: PMID 27348712

53. Rath S, Sharma R, Gupta R, Ast T, Chan C, Durham TJ, Goodman RP, Grabarek Z, Haas ME, Hung WHW, et al. MitoCarta3.0: An updated mitochondrial proteome now with sub-organelle localization and pathway annotations. Nucleic Acids Res 2021 ; 49(D1):D1541–7. Pubmed: PMID 33174596

54. Heberle H, Meirelles VG, da Silva FR, Telles GP, Minghim R. InteractiVenn: A web-based tool for the analysis of sets through Venn diagrams. BMC Bioinformatics 2015 ; 16(1):1–7. Pubmed: PMID 25994840

**Figure legends**

**Figure 1:** Differential degradation dynamics of mitochondrial sub-compartments correlate with delivery to the autophagolysosomal machinery. (**A**) Relative mitochondrial protein levels from Western blotting quantification. Data of at least three independent experiments. Data points: Mean ± SEM. (**B**) Representative immunofluorescence images of mitochondrial sub-compartments at the indicated CCCP treatment time points. Arrowheads indicate complete (4 h) or mitochondrial aggregates (14 h). Arrows indicate mitochondrial puncta. (**C**) TOM20-positive aggresomes (yellow) as well as SSBP1-aggregates (cyan) are positive for total ubiquitin (magenta) at the indicated time points. (**D and E**) TOM20 (magenta) and SSSBP1-aggresomes (cyan) are positive for (**D**) p62/SQSTM (yellow) and (**E**) TAX1BP1 at the indicated time points of CCCP treatment. (**F**) Mitochondrial puncta (TOM20, magenta and SSBP, cyan) co-staining with EGFP-LC3B (yellow). Arrows indicate mitochondrial puncta partially engaged with EGFP-LC3B structures. Closed arrowheads indicate complete overlap of mitochondrial puncta and EGFP-LC3B. Open arrowheads indicate EGFP-LC3B-negative mitochondrial puncta. Two ROIs are chosen in order to show all observed features: ROI 1 indicates a representative mitophagy stage for the 8 h time point. ROI 2 represents <10% of cell population where SSBP1 puncta can be observed already. (**G**) Representative immunofluorescence images showing TOM20 puncta (green) co-localizing with LysoTracker (magenta) at the indicated time points. Arrows indicate mitochondrial puncta while arrowheads indicate TOM20-aggregates. (**H and I**) Influence of BafA inhibition on OMM mitochondrial degradation. (**H**) Representative immunofluorescence images showing TOM20 (green) and SSBP1 (magenta) structures at the indicated CCCP-treatment time points in combination with autophagy inhibition (BafA). (**I**) Single-cell analysis of mean number of mitochondrial SSBP1- and TOM20 puncta in (**H**). Bars: Mean ± SEM. ****: p≤0.0001. All scale bars = 5 µm.

**Figure 2:** Autophagy activation and delivery to lysosomes of inner- mitochondrial material. (**A and B**) Influence of BafA inhibition on TOM20-negative mitochondrial-aggresomes. (**A**) Representative immunofluorescence images showing TOM20 (green) and SSBP1 (magenta) structures at the indicated CCCP-treatment time points in combination with autophagy inhibition (BafA). (**B**) Single-cell analysis of mean number of mitochondrial SSBP1 and TOM20 puncta in (**A**). (**C**) COX IV-positive puncta (magenta) are positive for EGFP-LC3B (yellow). Arrows indicate COX IV puncta partially engaged with EGFP-LC3B structures. Closed arrowheads indicate complete overlap of COX IV puncta and EGFP-LC3B. Open arrowheads indicate COX IV puncta non co-localizing with EGFP-LC3B. (**D**) Representative immunofluorescence images showing COX IV-positive puncta (green) co-localizing with LysoTracker (magenta) at the indicated time points. Arrows indicate mitochondrial puncta while arrowheads indicate inner- mitochondrial remnants. (**E and F**) Representative immunofluorescence images showing (**E**) COX IV-positive (magenta) or (**F**) SSBP1-positive mitochondrial remnants (magenta) close to LAMP1 patches (green) at the indicated time points. Arrows indicate mitochondrial puncta while arrowheads indicate inner- mitochondrial remnants. ****: p ≤0.0001. All scale bars = 5 µm.

**Figure 3:** Proteasome action is required at late stages of mitophagy. (**A**) Scheme of treatment time points for proteasome inhibition experiments. Bold text indicates treatment with CCCP while normal text indicates treatment with proteasome inhibitor MG132. (**B and C**) Western blot analysis of proteasome-influence on the indicated inner- mitochondrial proteins. (**D**) Representative images of WT-parkin HeLa cells showing SSBP1 (magenta) and TOM20 (green) after 24 h of CCCP treatment or CCCP treatment combined with MG132 added after 8 h of mitochondria depolarization. Scale bar = 5 µm. (**E and F**) Single cell analysis of mean SSBP1 clump number (**E**) and relative average SSBP1 clump area (**F**) per cell. Bars: Mean ± SEM. ns: non-significant; *: p≤0.05, ***: p≤0.001.

**Figure 4:** Proteomic validation of proteasome-sensitive steps during mitophagy. (**A**) Experimental design and dimethyl labelling scheme, where t defines the starting point of CCCP treatment. Color coding reflects dimethyl labelling sets. Solid: CCCP treatment, stripes: MG132 treatment. (**B**) Mitochondrial protein degradation after 8 h of mitophagy compared to cells pretreated with or without MG132 (t - 0.5 h) shows less degradation of OMM and IMS proteins. IMM and matrix proteins appeared less affected the 8 h time point as these sub-compartments were degraded during later stages of mitophagy. (**C**) Delayed MG132 (t + 8 h) treatment compared with not proteasome inhibited after 24 h of CCCP treatment highlights dependency of IMM and matrix protein degradation on proteasomal activity. The 5% and 10% curves in the upper panels categorize p≤0.05 and p≤0.1 values, respectively, for proteins with log2 T-test Difference >1.3.

**Figure 5:** Schematic representation of PINK1/parkin mitophagy dynamics. After healthy mitochondria are compromised with depolarizing agents, PINK1/parkin initial steps take place (1) and further promote mitochondrial network fractionation where a first round of proteasomal degradation is involved (2-3). Ubiquitinations previously built by parkin on OMM substrates promote the docking of autophagy adaptors (4) which engage with building autophagosomes that will eventually fuse with lysosomes, promoting autophagic degradation of OMM substrates (5-6). After the OMM removal, inner- mitochondrial aggregates prevail. A potential late-proteasome degradation step is required for the final degradation of inner- mitochondrial proteins before they can be engulfed in autophagolysosomes (7). Similar as in step (5), ubiquitinations of inner- mitochondrial substrates lead to the docking of autophagy adaptors and autophagosome biogenesis is promoted on those particular spots leading to the delivery of inner- mitochondria remnants to lysosomes for complete final mitochondria degradation (8-10). Adapted from “Cell Cycle Checkpoints” by BioRender.com (2022). Retrieved from <https://app.biorender.com/biorender-templates>.

**Supplementary Figure 1:** (**A and B**) Pretreatment with MG132 (**A**) of Bortezomib (**B**) impairs the elimination of immediate-proteasome degraded proteins as well as inner- mitochondrial proteins. (**C and D**) Early degradation of immediate-proteasomal proteins (green) in contrast to TOM20-positive mitochondrial aggregates (magenta) at the indicated time points. Representative images are shown for the average cell population and are indicated as ROI #1. ROI #2 represents <50% of cells showing slower mitophagy kinetics. (**E**) Endogenous LC3B structures (yellow) co-localize with TOM20 and COX IV puncta (magenta). Arrows indicate partial LC3B overlap with TOM20 or COX IV puncta. Closed arrowheads indicate complete LC3B overlap with both TOM20 puncta. (**F**) Representative images showing TOM20 (magenta) in close apposition with LAMP1 (green) at the indicated time points. Arrows indicate mitochondrial puncta in close apposition with LAMP1. All scale bars = 5 µm. (**G**) Mitochondrial markers on PentaKO cells transfected with Flag-parkin are not completely reduced after long exposure with CCCP treatment. (**H**) Western blot quantification of (**G**). (**I**) WT-parkin degradation kinetics upon CCCP treatment; replotted data from Figure 1A for side-by-side comparison. Data points are Mean ± SEM.

**Supplementary Figure 2:** (**A-F**) Single cell image quantification of mitochondrial profiles at early and late stages of mitophagy in WT-parkin expressing cells: (**A and B**) Mean number of complete mitochondria or mitochondrial remnants, identified by the indicated mitochondrial markers. (**C and D**) Relative cell area occupied by complete mitochondria or mitochondrial remnants, identified by the indicated mitochondrial markers. (**E**) Mean number of mitochondrial puncta per cell. (**F**) Relative cell area occupied by mitochondrial puncta. Bars: Mean ± SEM. ns: non-significant, *: p≤0.05, **: p≤0.005, ***: p≤0.001, ****: p≤0.0001. (**G and H**) Inner mitochondrial material is separate from TOM20 puncta. (**G**) Identification of TOM20-positive puncta (magenta) distinct from CS (green) or (**H**) COX IV (yellow) and SSBP1 (white) in HEK293 cells after 48 h of CCCP treatment. Arrowheads indicate TOM20-positive structures negative for inner- mitochondrial sub-compartment markers. (**I**) Identification of TOM20-positive puncta (magenta) distinct from CS (green) in two different NPC cell lines treated with antimycin A for 38 h. Arrowheads indicate TOM20-positive structures negative for inner- mitochondrial sub-compartment markers. (**J and K**) WT-parkin expressing cells (**J**) and HEK293 cells (**K**) pretreated with either DMSO or MG132 30 min before mitochondria depolarization was induced for the indicated time points. All scale bars = 5 µm.

**Supplementary Figure 3:** Example TEM images of autophagy-dependent mitochondrial turnover on WT-parkin HeLa. Examples of (**A**) elongated mitochondria (M) under basal conditions and (**B**) mitochondria engulfed by autophagosomes (A) (closed arrowheads) or mitochondrial aggresomes (right panel enlarged view of bracketed area) after 8 h CCCP treatment. (**C**) Autophagosome (A) engulfed mitochondria (M) after 4 h of CCCP treatment. Open arrowheads indicate broken OMM regions. Closed arrowheads indicate regions where OMM finishes abruptly.

**Supplementary Figure 4:** (**A-E**) Overlap of protein identification between replicates (n=3), with total number of identified proteins per replicate in brackets. Spearman rank correlation of log-transformed ratios between replicates. Representation for Set *blue* 8 h CCCP/ Ctrl. (**A**), Set *pink* 8 h CCCP, MG132 t - 0.5 h/ - (**B**), Set *pink* 8 h CCCP, MG132 t + 2 h/ - (**C**), Set *green* 24 h CCCP/ Ctrl. (**D**), Set *green* 24 h CCCP, MG132 t – 8 h/ Ctrl (**E**). (**F and G**) Significantly regulated proteins upon mitophagy induction for (**F**) 8 h or (**G**) 24 h. The 5% and 10% curves in the upper panels categorize p≤0.05 and p≤0.1 values, respectively, for proteins with log2 T-test Difference >1.3. Grey: total proteins, red: OMM, dark grey: IMS, blue: IMM, gold: matrix
