# Supplementary material for: Parkin-dependent mitophagy occurs via proteasome-dependent steps sequentially targeting separate mitochondrial sub-compartments for autophagy
Source: Autophagy Rep. 2022 Dec 19;1(1):576–602. doi: 10.1080/27694127.2022.2143214 (PMC11864704; doi:10.1080/27694127.2022.2143214)
Supplement: Supplemental Material [file KAUO_A_2143214_SM2097.zip › wetransfer_supplementary-figures_2022-11-22_0944.zip!/SupplementaryFigures_legends.docx]

**Supplementary Figure 1:** (**A and B**) Pretreatment with MG132 (**A**) or Bortezomib (**B**) impairs the elimination of immediate-proteasome degraded proteins as well as inner- mitochondrial proteins. (**C and D**) Early degradation of immediate-proteasomal proteins (green) in contrast to TOM20-positive mitochondrial aggregates (magenta) at the indicated time points. Representative images are shown for the average cell population and are indicated as ROI #1. ROI #2 represents <50% of cells showing slower mitophagy kinetics. (**E**) Endogenous LC3B structures (yellow) co-localize with TOM20 and COX IV puncta (magenta). Arrows indicate partial LC3B overlap with TOM20 or COX IV puncta. Closed arrowheads indicate complete LC3B overlap with both TOM20 puncta. (**F**) Representative images showing TOM20 (magenta) in close apposition with LAMP1 (green) at the indicated time points. Arrows indicate mitochondrial puncta in close apposition with LAMP1. All scale bars = 5 µm. (**G**) Mitochondrial markers on PentaKO cells transfected with Flag-parkin are not completely reduced after long exposure with CCCP treatment. (**H**) Western blot quantification of (**G**). (**I**) WT-parkin degradation kinetics upon CCCP treatment duplicated from Figure 1A for side-by-side comparison. Data points are mean: Mean ± SEM.

**Supplementary Figure 2:** (**A-F**) Single cell image quantification of mitochondrial profiles at early and late stages of mitophagy in WT-parkin expressing cells. (**A and B**) Mean number of complete mitochondria or mitochondrial remnants, identified by the indicated mitochondrial markers. (**C and D**) Relative cell area occupied by complete mitochondria or mitochondrial remnants, identified by the indicated mitochondrial markers. (**E**) Mean number of mitochondrial puncta per cell. (**F**) Relative cell area occupied by mitochondrial puncta. Bars: Mean ± SEM. ns: non-significant, *: p≤0.05, **: p≤0.005, ***: p≤0.001, ****: p≤0.0001. (**G and H**) Inner mitochondrial material is separate from TOM20 puncta. (**G**) Identification of TOM20-positive puncta (magenta) distinct from CS (green) or (**H**) COX IV (yellow) and SSBP1 (white) in HEK293 cells after 48 h of CCCP treatment. Arrowheads indicate TOM20-positive structures negative for inner- mitochondrial sub-compartment markers. (**I**) Identification of TOM20-positive puncta (magenta) distinct from CS (green) in two different NPC cell lines treated with antimycin A for 38 h. Arrowheads indicate TOM20-positive structures negative for inner- mitochondrial sub-compartment markers. (**J and K**) WT-parkin expressing cells (**J**) and HEK293 cells (**K**) pretreated with either DMSO or MG132 30 min before mitochondria depolarization was induced for the indicated time points. All scale bars = 5 µm.

**Supplementary Figure 3:** Example TEM images of autophagy-dependent mitochondrial turnover on WT-parkin HeLa. Examples of (**A**) elongated mitochondria (M) under basal conditions and (**B**) mitochondria engulfed by autophagosomes (A) (closed arrowheads) or mitochondrial aggresomes (right panel enlarged view of bracketed area) after 8 h CCCP treatment. (**C**) Autophagosome (A) engulfed mitochondria (M) after 4 h of CCCP treatment. Open arrowheads indicate broken OMM regions. Closed arrowheads indicate regions where OMM finishes abruptly.

**Supplementary Figure 4:** (**A-E**) Overlap of protein identification between replicates (n=3), with total number of identified proteins per replicate in brackets. Spearman rank correlation of log-transformed ratios between replicates. Representation for Set *blue* 8 h CCCP/ Ctrl. (**A**), Set *pink* 8 h CCCP, MG132 t - 0.5 h/ - (**B**), Set *pink* 8 h CCCP, MG132 t + 2 h/ - (**C**), Set *green* 24 h CCCP/ Ctrl. (**D**), Set *green* 24 h CCCP, MG132 t – 8 h/ Ctrl (**E**). (**F and G**) Significantly regulated proteins upon mitophagy induction for (**F**) 8 h or (**G**) 24 h. The 5% and 10% curves in the upper panels categorize p≤0.05 and p≤0.1 values, respectively, for proteins with log2 T-test Difference >1.3. Grey: total proteins, red: OMM, dark grey: IMS, blue: IMM, gold: matrix
